# Hearing Loss and Its Relation to Loneliness and Depression—A Population‐Based Cohort Study

**DOI:** 10.1002/lary.32060

**Published:** 2025-02-14

**Authors:** Julia Döge, Berit Hackenberg, Manfred E. Beutel, Daniëlle Otten, Philipp S. Wild, Julian Chalabi, GHS Research Consortium, Jasmin Ghaemi Kerahrodi, Thomas Münzel, Karl J Lackner, Irene Schmidtmann, Norbert Pfeiffer, Katharina Geschke, Peter R Galle, Christoph Matthias, Katharina Bahr‐Hamm

**Affiliations:** ^1^ Department of Otorhinolaryngology University Medical Center Mainz Mainz Germany; ^2^ Department of Psychosomatic Medicine and Psychotherapy University Medical Center Mainz Mainz Germany; ^3^ Center for Thrombosis and Hemostasis University Medical Center Mainz Mainz Germany; ^4^ DZHK (German Center for Cardiovascular Research), Partner Site RhineMain Mainz Germany; ^5^ Preventive Cardiology and Preventive Medicine – Department of Cardiology University Medical Center Mainz Mainz Germany

**Keywords:** depression, hearing aid, hearing impairment, hearing lossloneliness

## Abstract

**Objectives:**

Hearing loss is a prevalent factor contributing to reduced quality of life and has been linked to various comorbidities, with potential significant implications for psychosocial and cognitive health. The aim of this study was to give current information about the prevalence of hearing loss, loneliness and depression, and to examine the association between these.

**Study Design:**

Cohort study.

**Methods:**

The Gutenberg Health Study, a large representative cohort study for the general population, was initiated in 2007 at the University Medical Center Mainz, Germany. Hearing loss was assessed using pure‐tone audiometry, with severity graduated in percentages according to the World Health Organization (WHO). Loneliness and depressive symptoms were assessed using self‐report measures: Loneliness Scale (≥2) for loneliness and the PHQ‐9 (≥10) for depressive symptoms.

**Results:**

Among 5,948 participants (25–86 years), the prevalence of hearing loss was 38.7%. Loneliness was reported by 9.5% of the participants and the prevalence of significant symptoms of depression was 9.9%. The risk of loneliness was found to be significantly higher in participants with severe to complete hearing loss (OR = 3.92, *p* = 0.011). In addition, the odds ratio (OR) for depressive symptoms was significantly higher in those with a mild to severe hearing loss compared to those with normal hearing (OR = 1.3; *p* = 0.025). There was no association with hearing aid use.

**Conclusion:**

Hearing loss is associated with both loneliness and depressive symptoms. Longitudinal studies are required to clarify the causal relationships and to further investigate the direct impact of early hearing aid fitting on the progression of loneliness and depression.

**Level of Evidence:**

2 *Laryngoscope*, 135:2497–2505, 2025

## Graphical abstract

This study investigated the relationship between hearing loss, loneliness, and depression using data from the Gutenberg Health Study. It found that hearing loss is significantly associated with higher levels of loneliness and depressive symptoms, with severe to complete hearing loss markedly increasing risks for loneliness and mild to severe hearing loss for depressive symptoms. The study highlights the need for further longitudinal research to understand the causal mechanisms and the impact of early hearing aid use on these outcomes.

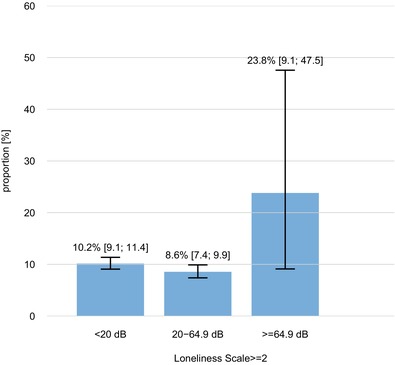

## INTRODUCTION

Globally, approximately 1.5 billion people suffer from some degree of hearing loss, and unaddressed hearing loss affects individuals of all ages, their families, and the economy.^1^ This number is expected to increase, making hearing loss a significant public health concern that can lead to a reduced quality of life, social isolation, and frustration.^2^ The prevalence of hearing loss increases with age and is higher among individuals with low socioeconomic status, but only some of those affected are provided with hearing aids.^3^ Research has established a link between hearing loss and loneliness, with potential implications for psychosocial and cognitive health.^4^ Despite the significance of this issue, representative and up‐to‐date data on the prevalence of objectively measured hearing loss and its association with loneliness and depression across various age groups remains limited.

In addition to the obvious challenges in communication, individuals with hearing loss often encounter various comorbidities that further diminish their quality of life. For example, numerous studies have demonstrated an association between hearing loss and dementia.^5^, ^6^ The 2020 Lancet Commission report identified hearing loss as the greatest modifiable risk factor for dementia in middle‐aged people compared to those without hearing impairment.^7^ In addition to an increased risk of dementia, patients with more severe hearing loss tend to exhibit more pronounced cognitive deficits.^8^ Social isolation and loneliness are hypothesized to be potential mechanisms linking hearing loss to declines in cognitive and mental health.^9^

Loneliness is typically defined as a subjectively negative perceived lack of social contact, belonging, or a sense of isolation.^10^ It refers to a discrepancy between desired and perceived social relations,^11^ either in quantity (referring to the number of social contacts) and/or the quality (indicating the subjective experience of characteristics such as affection, intimacy, or conflict).^12^ The literature has demonstrated a nonlinear age distribution for loneliness;^13^ it is particularly prevalent among young adults and among the oldest old.^14^ Although loneliness and social isolation are interrelated, they are distinct concepts. Social isolation refers to the objective state of having a limited social network and infrequent interactions with others, whereas loneliness is the subjective experience of this isolation. A person can be lonely without being socially isolated, and vice versa.^15^ Both, social isolation and loneliness are negatively associated with mental and physical health.^16^ Studies suggest that older individuals with hearing loss often encounter communication difficulties, which may lead to withdrawal from challenging situations and disrupt normal social behavior.^17^, ^18^ Shukla et al. have previously investigated the potential for modifying this hypothesized mechanistic pathway through the use of hearing aids.^4^

Depression is a highly prevalent risk factor for the onset of cardiovascular disease and is strongly associated with increased morbidity and mortality.^19^, ^20^ In Germany, the prevalence of depression rose to 15.7% in 2017, with females being twice as likely to be affected as males.^21^ Hearing loss appears to be associated with an increase in depressive symptoms,^22^ although the underlying mechanisms and causality remain unclear and underexplored, with mixed findings in the literature.^9^ Various methodological differences, such as differences in participant age, severity of hearing loss, and assessment of depression, likely contribute to that heterogeneity. Preliminary evidence suggests that audiological rehabilitation could be of great benefit for both hearing impairment and mental health.^22^, ^23^

The purpose of this study was to give current information about the prevalence of hearing loss, as objectively measured by pure‐tone audiometry, loneliness and depressive symptoms in a large population‐based cohort including various age groups in an adult population. Furthermore, hearing loss was correlated with loneliness and depressive symptoms.

## MATERIALS AND METHODS

The Gutenberg Health Study (GHS) is a large, ongoing population‐based cohort study. It was initiated in 2007 at Mainz University Medical Center in Germany. The population sample was randomly selected from the residents' registration office. Thus, the study population represents the people of Mainz and the district of Mainz‐Bingen. Approval was granted by the local institutional review board (Ethics Commission of the State Chamber of Physicians of Rhineland‐Palatine, reference no. 837.020.07) and was conducted in full compliance with the Declaration of Helsinki. At the 10‐year follow‐up (10‐FU, from 2017 to 2022) otologic examination was incorporated into the study's protocol. A detailed description of the study design has been published elsewhere.^24^ Only participants with available pure‐tone audiometry data were included in this study. Other exclusion criteria were missing data for loneliness and/or depression. This resulted in a total sample of *N* = 5,948.

Pure‐tone audiometry for both air‐ and bone‐conduction was performed separately for each ear.^25^ Hearing loss was then graded according to the World Health Organization (WHO) classification of hearing loss (mean value of the better hearing ear at the frequencies of 500, 1,000, 2,000, and 4,000 Hz).^26^ The prevalence of hearing loss was also weighted according to the German Standard Population 2021 (GSP) and to the European Standard Population 2013 (ESP).^27^ Three levels, according to the WHO,^1^ were created for statistical analyses:

- <20 dB HL: no hearing loss
- ≥20–64.9 dB HL: mild to moderately severe hearing loss
- ≥64.9 dB HL: severe to complete hearing loss.

Participants were asked “Are you fitted with hearing aids?” (yes/no).

Prevalence rates of loneliness (Loneliness Scale ≥2) and depressive symptoms (PHQ‐9 ≥10) were calculated.

Loneliness was assessed by the item, “I am frequently alone /have few contacts,” rated as 0 = no, does not apply, 1 = yes it applies, but I do not suffer from it, 2 = yes, it applies, and I suffer slightly, 3 = yes, it applies, and I suffer moderately, 4 = yes, it applies, and I suffer strongly. Loneliness was recoded combining 0 and 1 = no loneliness or distress; 2 = slight, 3 = moderate, and 4 = severe loneliness.^28^ Depression was assessed using diagnosis of depression and using the depression module of the Patient Health Questionnaire (PHQ‐9). With regard to diagnosis, participants were specifically asked the question, “Have you been diagnosed with depression by a doctor?” (yes/no). The PHQ‐9, which measures depressive symptoms based on the criteria outlined in the Diagnostic and Statistical Manual of Mental Disorders (DSM‐V),^29^ evaluates depressive symptoms experienced within the past 2 weeks. A total score of ≥10 out of 27 was considered as clinically relevant depressive symptoms.^30^

Socioeconomic status (SES) was calculated according to Lampert and Kroll.^31^ It has a sum value from 3 (lowest) to 21 (highest) and is formed from the characteristic's highest educational attainment, household income, and occupational position.

Continuous variables are presented as mean (standard deviation, SD) and tested with T‐test, or if |skewness| >1 as median (Q1, Q3) and tested with U‐test. Binary variables are described by relative and absolute frequencies and tested by chi‐square test. A multiple logistic regression model was conducted to test the likelihood of loneliness and depressive symptoms as a function of hearing loss. All analyses were of exploratory nature with p‐values (*P*) considered as continuous measure of statistical evidence.

All statistical analyses were performed in R version 4.1.0 (2021‐05‐18): R Core Team (2021).

## RESULTS

For the 10‐year follow‐up, 9752 participants took part in the follow‐up examination. Of these, 3,541 participants had missing or incomplete hearing data and were therefore excluded. Another 263 participants had missing data on loneliness or depression. Thus, the main cohort consisted of 5,948 participants. In this group of participants there were 3,061 males (51.5%) and 2,887 females (48.5%). The average age was 60.0 years (SD: 13.7; minimum and maximum: 25–86 years), with females having a slightly lower average age than males (females: 59.3 years [SD: 13.5], males: 60.6 years [SD: 13.8]).

The prevalence of hearing loss according to the WHO grading, including all levels was 38.7% (2,299/5,948). The distribution of hearing loss by severity was as follows: 26.6% of the cohort had mild hearing loss, 9.4% had moderate hearing loss, 2.2% moderately severe hearing loss, 0.3% severe hearing loss, 0.1% had profound hearing loss, and 0.1% exhibited complete hearing loss. See Table I and Appendix Table AI for the complete results. Weighted prevalence of hearing loss was lower at 28.4% according to GSP and 27.6% according to ESP.

**TABLE I:** Demographics and Prevalence of Hearing Loss, Loneliness, and Depressive Symptoms.

	All (*n*)	Men (*n*)	Women (*n*)	*p* value
*n*	5,948	3,061	2,887	
Average Age years (SD)	60.0 (13.7)	60.6 (13.8)	59.3 (13.5)	<0.001
Loneliness Scale	9.5% (457)	7.5% (185)	11.7% (272)	<0.001
≥2 (*n*)				
PHQ‐9 ≥ 10 (*n*)	9.9% (572)	7.2% (214)	12.7% (358)	<0.001
Depression, diagnosed (*n*)	5.5% (326)	3.8% (116)	7.3% (210)	<0.001
Use of hearing aids (*n*)		315	192	
Grades of hearing loss according to WHO, divided into three categories				
*N*	5,948			
<20 dB HL: no hearing loss	61.3% (3649)	57.1% (1748)	65.8% (1901)	
≥20–64.9 dB HL: mild to moderately severe hearing loss	38.2% (2,273)	42.4% (1,299)	33.7% (974)	
≥64.9 dB HL: severe to complete hearing loss.	0.4% (26)	0.5% (14)	0.4% (12)	
Hearing loss (HL ≥20 dB; both ears)	38.7% (2,299)	42.9% (1,313)	34.2% (986)	

Hearing loss increased significantly with age. The mean age difference is −16.6 years (95% CI: −17.18 to −16.1; *p* < 0.001). The difference refers to the group with no hearing impairment compared to the group with hearing impairment. Furthermore, the hearing ability of females was significantly better than that of males, with a mean difference of −9.2% (95% CI: −11.2% to −6.24%; *p* < 0.001) in the proportion of hearing loss, with the comparison made between the group with hearing impairment and the group without hearing impairment.

Approximately 7.8% of the participants had a hearing aid on the right ear and 8.1% had a hearing aid in the left ear. The reported prevalence of hearing aid provision (right and left) was significantly higher in males (*p* < 0.001).

A total of 9.5% of the participants reported loneliness (457/4786). In our cohort 4.4% were slightly, 3.6% moderately, and 1.5% severely distressed by loneliness. Significantly, more females than males felt lonely (females: 11.7%, males: 7.5%, *p* < 0.001).

Data on loneliness were available for the age groups ranging from 45 to 86 years.

The prevalence of significant symptoms of depression (PHQ‐9 ≥ 10) was 9.9% (572/5,786). A physician diagnosis was available for 5.5% of the participants (326/5,913). Females reported significantly more depressive symptoms than males (PHQ‐9 ≥ 10; females: 12.7%, males: 7.2%, *p* < 0.001) and were significantly more diagnosed with depression (females: 7.3%, males: 3.8%, *p* < 0.001). The proportion of participants with depressive symptoms decreased with age (*p* < 0.001). Table II shows the prevalence of hearing loss, loneliness, and depressive symptoms stratified according to sex and age.

**TABLE II:** Prevalence of Hearing Loss (HL ≥20 dB; Both Ears), Loneliness (Loneliness Scale ≥2), and Depressive Symptoms (PHQ‐9 ≥10; Stratified According to Sex and Age).

		Prevalence	Prevalence	Prevalence
		Hearing Impairment	Loneliness	Depressive Symptoms
	Age (Decades)	Proportion (*n*)	Proportion (*n*)	Proportion (n)
Men	25–34	2.0% (2)		10.8% (11)
	35–44	4.3% (14)		8.7% (28)
	45–54	11.5% (66)	10.7% (61)	7.5% (41)
	55–64	32.3% (241)	8.5% (63)	9.4% (68)
	65–74	63.0% (443)	5.9% (41)	5.4% (37)
	75–86	89.4% (547)	4.4% (20)	4.9% (29)
	Total	42.9% (1313)	7.5% (185)	7.2% (214)
Women	25–34	0.9% (1)		16.2% (18)
	35–44	4.2% (14)		14.8% (49)
	45–54	9.2% (56)	13.3% (80)	14.4% (85)
	55–64	25.2% (186)	13.4% (98)	16.2% (115)
	65–74	56.6% (360)	10.4% (66)	9.0% (56)
	75–86	80.2% (369)	7.9% (28)	7.8% (35)
	Total	34.2% (986)	11.7% (272)	12.7% (358)

Approximately 6.2% of the participants reported using antidepressant medication (367/5896). The following antidepressants were included in this category: nonselective monoamine reuptake inhibitors, selective serotonin reuptake inhibitors (SSRIs), nonselective monoamine oxidase inhibitors (MAOIs), monoamine oxidase A inhibitors (MAO‐A inhibitors), homeopathic and anthroposophic antidepressants, phytotherapeutic antidepressants, and other antidepressant medications.

**Fig. 1:**
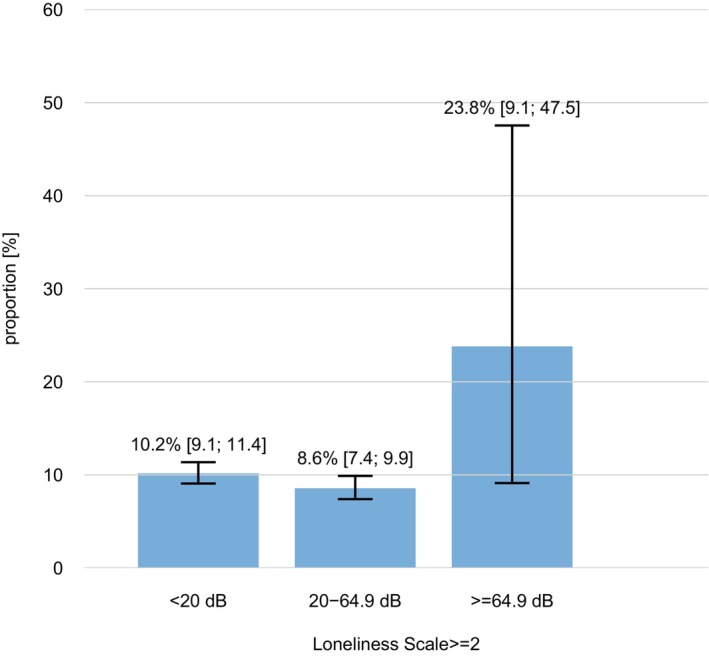
Prevalence of loneliness among participants by hearing levels (20 dB HL: no hearing loss, ≥20–64.9 dB HL: mild to moderately severe hearing loss, and ≥64.9 dB HL: severe to complete hearing loss). [Color figure can be viewed in the online issue, which is available at www.laryngoscope.com.]

**Fig. 2:**
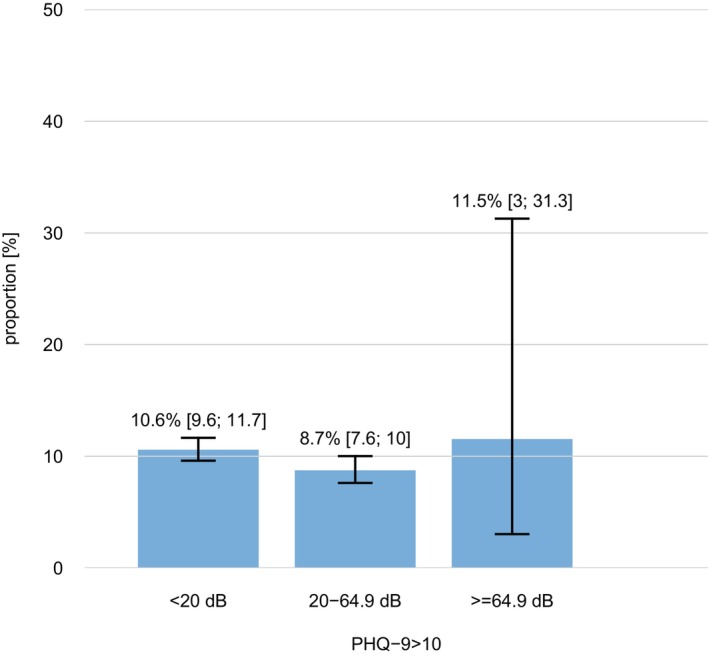
Prevalence of depressive symptoms among participants by hearing levels (20 dB HL: no hearing loss, ≥20–64.9 dB HL: mild to moderately severe hearing loss, and ≥64.9 dB HL: severe to complete hearing loss). [Color figure can be viewed in the online issue, which is available at www.laryngoscope.com.]

In the group with hearing loss (Hearing level ≥20 dB HL) the prevalence of loneliness was 8.7% compared to 10.2% among participants without hearing loss (Hearing Level < 20 dB HL; *p* = 0.1). Approximately 8.8% reported depressive symptoms in the group with hearing loss and among the group without hearing loss the prevalence of depressive symptoms was 10.6% (*p* = 0.027). The prevalence of diagnosed depression by a doctor as well as loneliness were higher for participants with severe to complete hearing loss (11.5% depression diagnosed vs. 5.7% within the group without hearing loss and 23.8% vs. 10.2%). Figure 1 shows the prevalence of loneliness and Figure 2 shows the prevalence of depression among participants by hearing levels.

**Fig. 3:**
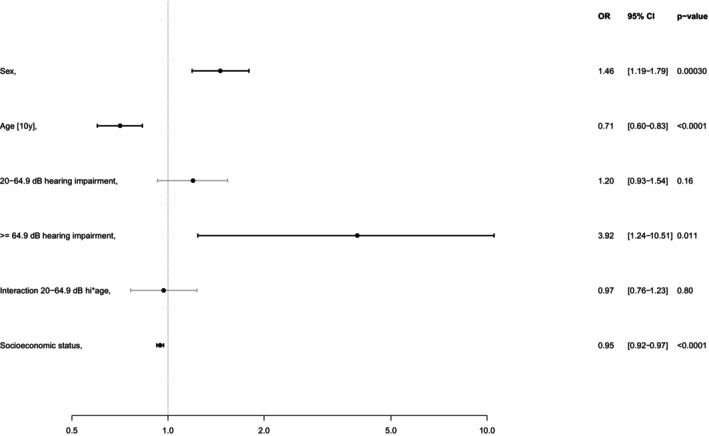
Logistic regression model for hearing loss and loneliness by hearing levels (reference is hearing level HL ≤20 dB; interaction term age was added). OR = odds ratio, CI = confidence interval.

**Fig. 4:**
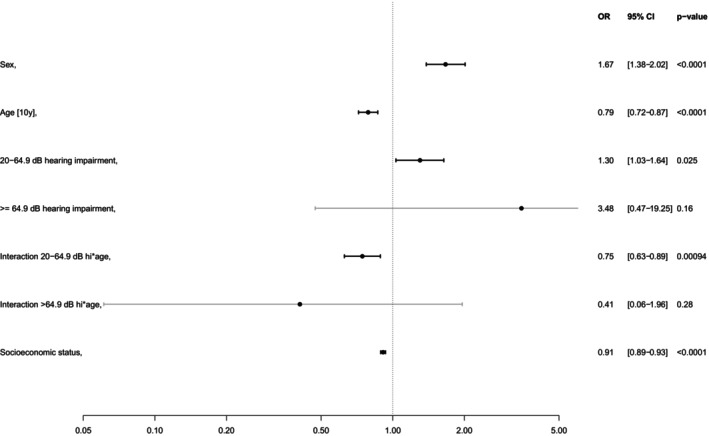
Logistic regression model for hearing loss and depressive symptoms by hearing levels (reference is hearing level HL ≤20 dB; interaction term age was added). OR = odds ratio, CI = confidence interval.

Logistic regression results showed that severe to complete hearing loss significantly increases the odds of loneliness (OR = 3.92, 95% CI [1.24–10.51], *p* = 0.011; for more details see Fig. 3). The likelihood of depressive symptoms was significantly higher for mild to moderately severe hearing loss (OR = 1.3, 95% CI [1.03–1.64], *p* = 0.025; see Fig. 4). As age has a significant influence, an interaction term was added for the mild to moderate severe hearing loss group. Within this group, the risk of depression decreases significantly with the average age (*p* < 0.001). There was no significant association with the use of hearing aids (*p* = 0.18 for loneliness and *p* = 0.92 for depression). Higher socioeconomic status was significantly associated with lower levels of both loneliness and depression (*p* < 0.001). Additionally, a comorbidity index was constructed as a sum score based on six variables: chronic obstructive pulmonary disease, coronary artery disease, peripheral artery disease, atrial fibrillation, myocardial infarction, stroke. The regression models were adjusted for this index. The adjustment did not affect other effects we reported.

## DISCUSSION

Current and representative data on the prevalence of objectively measured hearing loss and its association with loneliness and depression across various age groups are limited. Therefore, this study aimed to examine the prevalence of objectively measured hearing loss, loneliness and depressive symptoms, as well as the correlations among these factors, within a large population‐based cohort among adults.

Among the 5,948 participants, the prevalence of hearing loss was 38.7%. Loneliness was reported by 9.5% of participants, while 9.9% exhibited depressive symptoms. The odds ratio (OR) for loneliness was significantly elevated in participants with severe to complete hearing loss (OR = 3.92; *p* = 0.011). Participants with mild to severe hearing loss had a significantly higher risk of depression (OR = 1.3; *p* = 0.025). The main finding of this study is that hearing loss is associated with both loneliness and depression. Moreover, higher socioeconomic status had a positive effect on both loneliness and depression.

In our study, the prevalence of hearing loss was high, exceeding 38%. In this context, hearing loss among older adults represents a substantial burden of disease.^32^ Some studies have shown that older people with hearing loss experience communication difficulties, leading to frustration and withdrawal.^33^ This can contribute to social isolation^34^ and a diminished quality of life.^35^, ^36^ Beyond its impact on mental health, hearing loss is also associated with increased risks of total mortality and heart disease mortality.^37^ Regular use of hearing aids appears to be associated with a lower risk of mortality in adults with hearing loss compared to nonusers.^38^

In their review, Shukla et al. identified 11 studies that examined the association between hearing loss and loneliness in older adults.^4^ The studies exhibited heterogeneity in their assessment of both loneliness and hearing loss. Of the nine cross‐sectional studies, six found an increase in loneliness among older adults with hearing loss, while one of two longitudinal studies reported similar findings. These results partially align with our study, as we found positive associations between severe hearing loss and loneliness. Consistent with our findings, Sung et al. reported that younger age and a greater hearing loss were significantly associated with greater loneliness.^39^ Three studies, however, found no association between hearing loss and loneliness, though two of these relied on unadjusted crude analyses. It is important to note that older individuals may not necessarily perceive an objective reduction in social engagement as loneliness. The scoping review by Bott et al. concluded that hearing loss is associated with both social isolation and loneliness, regardless of the degree of hearing loss.^40^ This is consistent with our results, where we found that severe to complete hearing loss more than tripled the odds of loneliness (OR = 3.92, *p* = 0.011). Although the likelihood of loneliness was also higher in participants with mild to moderately severe hearing loss, it was not statistically significant (OR = 1.2, *p* = 0.16). This lack of significance may be attributed to the relatively low perceived communication difficulties associated with mild hearing loss.

In their review, Ellis et al. identified three studies examining the effects of hearing interventions on loneliness among older people.^41^ Two of the studies used the De Jong Gierveld (DG) Loneliness Scale involving hearing aid fittings as the intervention in both the studies. Weinstein et al. found a significant reduction in overall loneliness.^42^ Similarly, Sarant et al. observed a decrease in the number of participants experiencing severe loneliness, although statistical analysis was limited by the small sample size.^43^ Additionally, a study using the UCLA Loneliness Scale found a significant reduction in loneliness following cochlear implantation.^44^ Marques reported that hearing aid use is associated with improvements in depressive symptoms and can enhance overall quality of life in older adults.^22^ In our study, we did not observe a significant effect of hearing aids on loneliness or depression. It is important to note, however, that only a small proportion of the participants with a hearing loss were fitted with hearing aids, leading to a limited sample size for this subgroup. Furthermore, our study was not an intervention study.

Bott et al. suggested that the impact of hearing loss on health and well‐being may vary with age.^40^ In our study, which included a large cohort spanning various age groups, we found a significant association between age, sex, and the occurrence of depressive symptoms. Specifically, the prevalence of depressive symptoms decreased with increasing age, and females reported significantly higher levels of depressive symptoms compared to males. Conversely, the proportion of participants with hearing loss increased with age, while females, on average, had better hearing than males. This difference may contribute to the observed higher prevalence of depressive symptoms among younger participants, who generally had no hearing loss, compared to those with mild to severe hearing loss (see Fig. 2).

Rutherford et al. reported mixed results regarding the association between hearing loss and late‐life depression, suggesting that there was an association.^9^ They noted that high‐quality studies with objective hearing assessments are scarce and exhibit considerable heterogeneity in their findings. Similarly, Cosh et al. reported an association between hearing loss and clinically significant depressive symptoms; however, heterogeneity between studies arises due to methodological differences, including age, severity of hearing loss, and assessment of depression.^23^ The systematic review from Lawrence et al. found that hearing loss was associated with a 1.47 times higher likelihood of depression in older adults (≥60 years), though the effect size was small. The relationship did not appear to be affected by the type of hearing loss measurement, use of hearing aids, or demographic and health factors.^45^

Our study benefits from the use of standardized pure‐tone audiometry and represents the first study of this scale conducted in Germany. Our data indicate that mild to moderately severe hearing loss significantly, positively predicted depressive symptoms (OR = 1.3; *p* = 0.025). Given the substantial influence of age on depressive symptoms, an interaction term was added in our logistic regression model, which revealed that the risk of depressive symptoms significantly decreases with increasing age within the hearing loss group. This finding may help explain why participants with severe to complete hearing loss, which is more prevalent with advancing age, do not exhibit a significantly higher risk of depression. Our results are consistent with those of Gopinath et al., who found that depressive symptoms were associated with mild but not moderate or severe hearing loss in a prospective, cross‐sectional study involving 1,328 participants aged 60 and older.^46^ Furthermore, there appears to be no significant difference between the right and left ears; deterioration in hearing in either ear is associated with an increase in depressive symptoms.^47^

A major strength of the study is its large sample size and population‐based design. The GHS achieved representative results with data from 5,948 participants across various age groups in an adult population. Additionally, high‐quality tone‐audiometric data were collected using pure‐tone audiometry, and standardized tests were also used to assess loneliness and depressive symptoms. However, a limitation of this study is the unresolved issue of causality regarding the association between hearing loss and loneliness and depressive symptoms. Moreover, we lack information on social isolation, which has been frequently linked to loneliness in the literature. Furthermore, the number of participants using hearing aids and those with severe to profound hearing loss is relatively small, and there is no longitudinal assessment of changes in loneliness and depression following hearing aid fitting.

## CONCLUSION

This study demonstrates a positive association between hearing loss and both loneliness and depressive symptoms. Given the widespread prevalence of hearing loss, which is expected to increase, this poses significant implications for the psychosocial and physical health of individuals, particularly older adults, and represents a major public health concern. Future research should focus on elucidating the causal relationships involved and evaluating the direct impact of early hearing aid intervention on the progression of loneliness and depression.

## GHS Research Consortium

Jasmin Ghaemi Kerahrodi MD; Thomas Münzel MD, PhD; Karl J Lackner MD, PhD; Irene Schmidtmann PhD; Norbert Pfeiffer MD, PhD; Katharina Geschke MD; Peter R Galle MD, PhD.

## Appendix

**TABLE AI:** Prevalence of Hearing Loss According to the World Health Organization Grading, Stratified by Sex.

Grades of hearing loss according to WHO	All (n)	Men (n)	Women (n)
*n*	5,948	3,061	2,887
Normal hearing	61.3% (3,649)	57.1% (1,748)	65.8% (1,901)
Mild hearing loss	26.6% (1,583)	28.8% (881)	24.3% (702)
Moderate hearing loss	9.4% (562)	10.8% (332)	8.0% (230)
Moderately severe hearing loss	2.2% (128)	2.8% (86)	1.5% (42)
Severe hearing loss	0.3% (15)	0.2% (6)	0.3% (9)
Profound hearing loss	0.1% (3)	0.1% (2)	0% (1)
Complete or total hearing loss	0.1% (8)	0.2% (6)	0.1% (2)
Hearing loss (HL ≥20 dB; both ears)	38.7% (2,299)	42.9% (1,313)	34.2% (986)
